# Reduction of oxidative-nitrosative stress underlies anticataract effect of topically applied tocotrienol in streptozotocin-induced diabetic rats

**DOI:** 10.1371/journal.pone.0174542

**Published:** 2017-03-28

**Authors:** Nurul Alimah Abdul Nasir, Renu Agarwal, Siti Hamimah Sheikh Abdul Kadir, Sushil Vasudevan, Minaketan Tripathy, Igor Iezhitsa, Aqil Mohammad Daher, Mohd Ikraam Ibrahim, Nafeeza Mohd Ismail

**Affiliations:** 1 Center for Neuroscience Research, Faculty of Medicine, Universiti Teknologi MARA Sungai Buloh Campus, Sungai Buloh, Selangor, Malaysia; 2 Faculty of Pharmacy, Universiti Teknologi MARA Puncak Alam Campus, Puncak Alam, Selangor, Malaysia; 3 Research Institute of Pharmacology, Volgograd State Medical University, Volgograd, Russia; 4 Department of Community Medicine, Faculty of Medicine and Defence Health, National Defence University of Malaysia, Sungai Besi Camp, Kuala Lumpur, Malaysia; Shiraz University, ISLAMIC REPUBLIC OF IRAN

## Abstract

Cataract, a leading cause of blindness, is of special concern in diabetics as it occurs at earlier onset. Polyol accumulation and increased oxidative-nitrosative stress in cataractogenesis are associated with NFκB activation, iNOS expression, ATP depletion, loss of ATPase functions, calpain activation and proteolysis of soluble to insoluble proteins. Tocotrienol was previously shown to reduce lens oxidative stress and inhibit cataractogenesis in galactose-fed rats. In current study, we investigated anticataract effects of topical tocotrienol and possible mechanisms involved in streptozotocin-induced diabetic rats. Diabetes was induced in *Sprague Dawley* rats by intraperitoneal injection of streptozotocin. Diabetic rats were treated with vehicle (DV) or tocotrienol (DT). A third group consists of normal, non-diabetic rats were treated with vehicle (NV). All treatments were given topically, bilaterally, twice daily for 8 weeks with weekly slit lamp monitoring. Subsequently, rats were euthanized and lenses were subjected to estimation of polyol accumulation, oxidative-nitrosative stress, NFκB activation, iNOS expression, ATP levels, ATPase activities, calpain activity and total protein levels. Cataract progression was delayed from the fifth week onwards in DT with lower mean of cataract stages compared to DV group (p<0.01) despite persistent hyperglycemia. Reduced cataractogenesis in DT group was accompanied with lower aldose reductase activity and sorbitol level compared to DV group (p<0.01). DT group also showed reduced NFκB activation, lower iNOS expression and reduced oxidative-nitrosative stress compared to DV group. Lenticular ATP and ATPase and calpain 2 activities in DT group were restored to normal. Consequently, soluble to insoluble protein ratio in DT group was higher compared to DV (p<0.05). In conclusion, preventive effect of topical tocotrienol on development of cataract in STZ-induced diabetic rats could be attributed to reduced lens aldose reductase activity, polyol levels and oxidative-nitrosative stress. These effects of tocotrienol invlove reduced NFκB activation, lower iNOS expression, restoration of ATP level, ATPase activities, calpain activity and lens protein levels.

## Introduction

Cataracts are one of the common causes of visual impairment in diabetic subjects [1, 2]. In patients with diabetes, cataract has an early onset and develops 2 to 5 times more frequently [3–5]. Development of cataractous opacities in diabetics is a consequence of alterations in several metabolic pathways due to long standing hyperglycemia that culminate into increased lens oxidative and nitrosative stress and impaired functions of enzymes that maintain lens water and electrolyte homeostasis [6, 7].

Among the hyperglycemia-triggered metabolic changes, polyol pathway in which aldose reductase (AR) is the first enzyme involved, is widely investigated [8, 9]. AR converts glucose into sorbitol, a polyol, and utilizes NADPH. Highly active polyol pathway depletes NADPH, which is also required as cofactor for synthesis of reduced glutathione (GSH), hence reducing GSH synthesis [10]. Additionally, non-enzymatic glycation of antioxidant enzymes impairs their function and further contributes to ROS generation and oxidative stress. Increased availability of ROS promotes its reaction with nitric oxide (NO) to form peroxynitrite (ONOO^-^), a potent reactive nitrogen species. This reaction occurs almost five times faster compared to the neutralization rate of superoxide radicals by superoxide dismutase (SOD), hence favoring ONOO^-^ formation [11]. In lenticular cells, excessive NO formation results from increased inducible nitric oxide synthase (iNOS) expression [12], secondary to activation of nuclear factor kappa B (NFκB) [13, 14]. ONOO^-^ also causes sustained NFκB activation by favouring its release from its complex with inhibitory kappa B (IκB) [15]. High level of NO associated with increased iNOS expression have been observed in cataractous lenses [16,17].

Increased oxidative stress also causes cellular ATP depletion due to mitochondrial dysfunction [18–21]. Cellular ATP loss and non-enzymatic glycation result in impaired function of ATPases such as Na^+^ K^+^ ATPase and Ca^2+^ ATPase [22,23]. Na^+^ K^+^ ATPase regulates sodium, potassium and water homeostasis in the lens, and its reduced functions lead to intracellular accumulation of sodium and water [24]. Similarly, impaired functions of plasma membrane Ca^2+^ ATPase (PMCA) and sarcoplasmic/endoplasmic reticulum Ca^2+^ ATPase (SERCA), which regulate the intracellular calcium homeostasis [25], lead to high intracellular calcium and extremely high calcium level has been detected in cataractous lenses compared to normal lenses [26]. High intracellular calcium activates the calcium-dependent cysteine protease, calpain, which was shown to promote crystallin proteolysis, leading to opacification of the lens [27–29]. Crystallins are the proteins essential for lens transparency and change in their structure leads to opacification of the lens [30].

Our previous studies have shown that topical application of the microemulsion of tocotrienol, a vitamin E analog, delays the onset and progression of galactose-induced cataract in rats and the maximum anticataract efficacy was observed at a concentration of 0.03%. Furthermore, this anticataract effect was associated with reduced lenticular oxidative and nitrosative stress [31]. However, the mechanisms underlying these effects of tocotrienol remain unclear. Hence, in this study we used streptozotocin (STZ)-induced rat model of diabetes, which is a closer representation of human diabetic cataract, to investigate the effects of topical application of tocotrienol on lenticular polyol pathway, NFκB activity, expression of iNOS, oxidative stress levels, ATP, ATPases, calpain 2 activities and protein levels. We correlated these effects of tocotrienol with changes in lenticular oxidative-nitrosative stress and progression of cataract.

## Materials and methods

### Animals

Animal handling and all procedures were performed in line with ARVO statement for the use of animals in ophthalmic and vision research as well as local institutional ethical guidelines by Animal Care & Use Committee (ACUC), Faculty of Medicine of Universiti Teknologi MARA under approval number ACUC-9/13. Male *Sprague-Dawley* rats weighing 150–200 g were obtained from Laboratory Animal Care Unit (LACU) of Universiti Teknologi MARA and maintained under standard laboratory conditions at 12 hours light/dark cycle. Animals were caged individually and given food and water ad libitum. All animals were subjected to systemic and ophthalmic examination and those found normal were included in the study. Since diabetic animals had polyuria, bedding was changed at least twice a day to ensure cleanliness and hence minimize the risk of infection. All animals were monitored daily for their wellbeing.

### Study design

A pilot study was initially performed to evaluate effect of vehicle on the lenticular transparency in STZ- induced diabetic rats. The vehicle later was used to formulate tocotreinol microemulsion for further studies. Animals were divided into three groups (n = 6 per group). Group 1 received sodium citrate buffer intraperitoneally (NP) whereas groups 2 (SP) and 3 received intraperitoneal STZ. Post 48 hours of STZ injection, group 3 (VP) was treated with a single 10 μl drop of vehicle topically, bilaterally, twice daily for 8 weeks. Anterior segment imaging was done weekly to assess the lenticular changes. Blood glucose and body weight were also monitored weekly.

For the main experimental study, animals were divided into three groups (n = 39; 78 eyes per group). Group 1 consisted of rats that received sodium citrate buffer intraperitoneally. These rats were treated with vehicle (NV). Animals in groups 2 and 3 received intraperitoneal STZ and were treated with vehicle (DV) and 0.03% microemulsion of tocotrienol (DT), respectively. All treatments started 48 hours postintraperitoneal injections and were given topically in a volume of 10 μl, bilaterally, twice daily for 8 weeks. The choice of 0.03% concentration of tocotrienol was based on our previous study [31].

Anterior segment imaging was done once before intraperitoneal injections as a baseline and, subsequently, weekly post-STZ/ sodium citrate buffer injection. Blood glucose level and body weight were recorded weekly during the experimental period. After eight weeks of treatment, the animals were sacrificed with overdose of intraperitoneal ketamine (250 mg/kg) and xylazine (50 mg/kg). The lenses were carefully dissected out and stored at -80°C until further analysis. The lenses were processed for estimation of lens polyol contents, oxidative-nitrosative stress, NFκB signaling pathway, ATP contents and ATPase activities, calpain 2 activity and proteins levels. Generally, each lens was homogenized in 1 ml of 50 mM cold phosphate buffered saline (PBS, pH 7.4, with 1 mM EDTA), unless stated otherwise. The homogenate was centrifuged at 890 g for 15 min. Supernatant was separated and used for estimation of the biochemical parameters. All estimates were done in duplicate.

### Microemulsion formulation of tocotrienol

Microemulsion formulation of tocotrienol was prepared as described previously by Nasir et al [31]. Briefly, Kolliphor P188 (Sigma Aldrich, St. Louis, MO) was added into double distilled water to create an aqueous phase. Annatto tocotrienol, which contained 90% of δ-tocotrienol, 10% of γ-tocotrienol and no tocopherol, was a gift from American River Nutrition, Inc. (Hadley, MA). Tocotrienol was added into Miglyol 812 (AXO Industry, Wavre, BE) to create an oily phase. The oily phase was then added into the aqueous phase under moderate agitation. Subsequently, the particle size was reduced using ultrasound sonicator (Fisher Scientific, FB120, Hampton, NH) for 40 minutes at the setting of 80% amplitude with cycles of 50 seconds on and 20 seconds off. After sonication, sorbitol and disodium edetate (EDTA, 0.1%) (Fisher Chemical, Hampton, NH) were added as isotonizing agent and stabilizer respectively. Vehicle microemulsion was formulated as above without the addition of tocotrienol in the oily phase.

### Induction of diabetes with STZ

Animals were fasted overnight and then were given intraperitoneal injection of 65 mg/kg STZ in sodium citrate buffer (10 mmol/L, pH 4.5). Forty-eight hours post-STZ injection, blood was collected from tail vein for determination of blood glucose level using Accu Chek Performa glucometer (Roche Diagnostic, Basel, CH). Animals with blood glucose level more than 20 mmol/L were included for further study. Control rats were similarly injected with sodium citrate buffer that was used as vehicle for STZ.

### Anterior segment imaging

**Fig 1:**
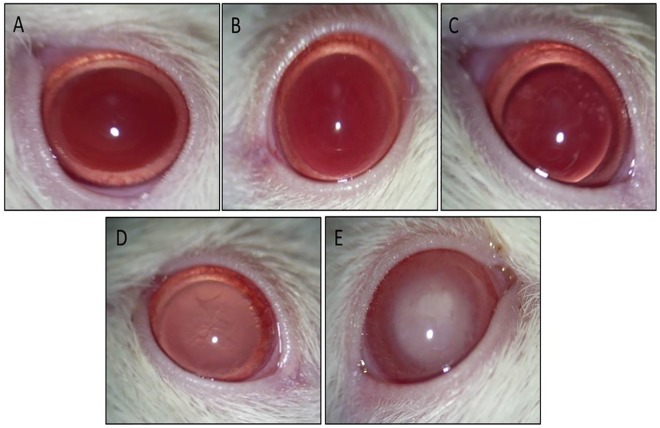
Retroillumination anterior segment photographs showing progression of cataract from stage 0 to stage 4 in STZ-induced diabetic rats. A: Stage 0. B: Stage 1. C: Stage 2. D: Stage 3. E: Stage 4.

Anterior segment imaging was done using Hawkeye Portable Slit Lamp (Optotek Medical, Ljubljana, SI) equipped with digital camera (Pentax Optio, S60, Denver, CO). Tropicamide 1% (Alcon Laboratories, Inc., Fort Worth, TX)) was topically applied 1 minute prior to imaging for mydriasis. Animals were lightly restrained with hand to get a still lens image. Lenticular changes were graded as described by Suryanarayana et al. [32] with slight modification. Accordingly, changes were categorized into 4 stages: Stage 0—normal lenses; Stage 1 –minimal opacity at the centre of lens; Stage 2 –patchy appearance of opacity both in the centre and periphery of lens; Stage 3—uniform opalescence all over the lens; Stage 4 –mature cataract with nuclear opacity (Fig 1). The grading of cataractous changes was done independently by three observers that were unaware of the grouping of animals.

### Quantification of lens polyol contents and AR activity

Extent of polyol accumulation in lenses was estimated by measuring lens D-sorbitol concentration using Colorimetric Assay Kit (Biovision, CA, USA) and AR activity using sandwich-ELISA kit (Elabscience, Wuhan, China) as per manufacturer’s instructions.

### Estimation of the lenticular NFκB expression and activation

Expression of NFκB was first visualized by immunohistochemistry (IHC). For quantitative estimation of the extent of NFκB activation, we measured cytoplasmic levels of phosphorylated IκB-α (pIκB-α) in the cytoplasmic extract and NFκB p65 in the nuclear extract.

For immunohistochemistry, lenses were directly immersed in 10% buffered formalin after washing with PBS and then were processed for paraffin embedding. Subsequently, eyes were sectioned at a thickness of 4 μm with rotary microtome. Tissue sections were deparaffinized with xylene and alcohol and then antigen retrieval was done by heating the sections in 10 mM citrate buffer (pH 6.0) for 8 min at 95°C. Staining was done using Pierce^®^ Peroxidase Detection Kit (Pierce Biotechnology, Rockford, IL, USA). After antigen retrieval, tissues were incubated with hydrogen peroxide for 30 minutes and washed. Then, non-specific binding of antibody was blocked using blocking buffer for 30 minutes and incubation was done with primary antibody (NFκB p65 rabbit polyclonal antibody, 1:100, Pierce Biotechnology, Rockford, IL, USA) for 30 minutes. After washing, tissue sections were incubated with horseradish peroxidase (HRP) conjugated secondary antibody (Ready-to-Use Goat anti rabbit/mouse antibody, Dako, Glostrup, Denmark) for another 30 minutes. The immunostaining was performed using 3,3’-diaminobenzidine (DAB)/ metal and peroxide mixture for 10 minutes. Tissue sections were then counterstained with hematoxylin for 2 minutes, dehydrated and mounted. All washes in this procedure were done three times for 3 minutes each with TBS mixed with 10% Tween 20 (TBST), whereas antibodies were diluted in blocking buffer. For negative controls, tissue sections were incubated with TBST.

For quantification of NFκB and pIκBα activities, extraction of lenticular nuclear and cytoplasmic fraction was done using NE-PER Nuclear and Cytoplasmic Extraction Reagent Kit (Thermo Scientific, Rockford, IL). Two lenses were pooled together as one sample. Lenses were cut into small pieces and washed with ice-cold PBS followed by centrifugation at 890 g for 5 minutes at 4°C. The supernatant was discarded and the remaining lens tissue pellet was homogenized with cytoplasmic extraction reagent I (CER I) at a ratio of 1 mg lens tissue: 10 μl CER I using a tissue grinder on ice. Then, tissue suspension was vortexed at highest setting for 15 seconds and incubated on ice for 10 minutes. Cytoplasmic extraction reagent II (CER II) was added to the tissue suspension at the ratio of 1 mg lens tissue: 0.55 μl CER II and vortexed for 5 seconds. After 1 minute incubation on ice, the suspension was vortexed again at highest setting for 5 seconds and then, centrifuged at 11900 g for 5 minutes at 4°C. The supernatant which contained cytoplasmic extract was used to measure pIκB-α level. The remaining tissue pellet was resuspended with the provided nuclear extraction reagent (NER) at the ratio of 1 mg lens tissue: 5 μl NER. Tissue suspension was vortexed at highest setting for 15 seconds and incubated on ice for 10 minutes and this cycle of vortex and ice incubation was continued for a total of 40 minutes. Then, tissue suspension was centrifuged at 11900 g for 10 minutes at 4°C. The supernatant which contained nuclear extract was collected for measurement of NFκB. Both the pIκB-α and NFκB estimations were done using commercially available sandwich-ELISA kits (Elabscience, Wuhan, China) containing wells pre-coated with antibodies specific to pIκB-α and NFκB, respectively, as per manufacture’s instruction.

### Estimation of iNOS expression

Quantitative estimation of iNOS protein expression was done using commercially available sandwich-ELISA kit (Elabscience, Wuhan, China) containing wells pre-coated with antibody specific to iNOS, as per manufacturer’s instructions. All estimations were done in duplicate. In addition, gene expression for *iNOS* mRNA was assessed using qPCR.

Lens tissue was placed in RNAlater Stabilization Solution directly after dissection to stabilize and protect cellular RNA. RNA extraction and purification was done using GeneJET RNA purification kit (Thermo Scientific Inc., Rockford, IL). The extracted RNA was then cleaned up to remove any genomic DNA present using RapidOut DNA Removal Kit (Thermo Scientific Inc., Rockford, IL). RNA concentration was determined using Nanodrop Spectrophotometer 1000 (Thermo Scientific Inc., Rockford, IL), whereas, RNA quality was assessed using Agilent RNA 6000 Nano kit (Agilent Technologies, Santa Clara, CA) based on the RNA integrity number (RIN) read by Agilent 2100 Bioanalyzer instrument (Agilent Technologies, Inc., Santa Clara, CA). Then, cDNA synthesis was performed using Maxima First Strand cDNA Synthesis Kit (Thermo Scientific Inc., Rockford, IL). Briefly, 1μg RNA was added to 5X Reaction Mix (contained reaction buffer, dNTPs, oligo (dT)18 and random hexamer primers), reverse transcriptase, RNase inhibitor and nuclease free water to make a solution volume up to 20 μl. The solution was gently mixed and centrifuged for 1 minute and then incubated at 25°C for 10 minutes, followed by 50°C for 15 minutes and lastly at 85°C for 5 minutes.

*iNOS* gene expression was measured in relation to expression of two reference genes; glyceraldehyde- 3-phosphate dehydrogenase (*GADPH*) and β- actin. The primers used were; 5′-CACGGCAAGTTCAACGGCACAG-3′ and 5′-ACGCCAGTAGACTCCACGACAT-3′ for *GADPH*, 5′-ACTCTTCCAGCCTTCCTTC-3′ and 5′-ATCTCCTTCTGCATCCTGTC-3′ for *β- actin*, 5′-CTTGGAGCGAGTTGTGGATTGT-3′ and 5′-GTAGTGATGTCCAGGAAGTAGGT-3′ for iNOS. qPCR reaction was performed with Luminaris Color HiGreen qPCR Master Mix (Thermo Scientific., Inc, Rockford, IL) using CFX96 Real Time System (Bio-Rad, Hercules, CA, USA). The following PCR cycling conditions were used for both GADPH and β- actin primer: 120 seconds of uracil-DNA glycosylase (UDG) pretreatment at 50°C, followed by 180 seconds of initial denaturation at 95°C, followed by 50 cycles of 30 seconds at 95°C, 30 seconds at 60°C. Whereas, for *iNOS*, the following cycling conditions were used: 120 seconds of UDG pretreatment at 50°C, followed by 180 seconds of initial denaturation at 94°C, followed by 55 cycles of 30 seconds at 94°C, 30 seconds at 62°C. Cycle threshold (Ct) values were measured and calculated by the CFX software (version Bio-Rad CFX Manager 2.0).

### Quantification of lens oxidative and nitrosative stress

Lens oxidative stress was measured by estimation of malondialdeyhde (MDA), GSH contents, SOD and catalase (CAT) activities in lens tissue. For all oxidative stress parameters, commercially available ELISA kits (Cayman Chemicals, Ann Arbor, MI) were used and all estimations were done in duplicate. TBARS Assay kit indirectly measures MDA, a byproduct of lipid peroxidation. MDA reacts with thiobarbituric acid (TBA) under high temperature and acidic medium to produce coloured complex. For this assay, lenses were homogenized with RIPA lysis buffer containing protease inhibitor in a ratio of 1 mg lens weight: 10 μl RIPA buffer. Samples were then centrifuged at 890 g at 4°C for 10 minutes and supernatant was used for analysis. Measurement of SOD activity was based on utilization of tetrazolium salt for detection of superoxide radicals generated by xanthine oxidase and hypoxanthine. Estimation of lens CAT activity was based on the CAT reaction with methanol in the presence of the optimal H_2_O_2_ concentration. The production of formaldehyde was measured by adding 4-amino-3-hydra¬zino-5-mercapto-1, 2, 4-triazole, a chromagen (purpald). Purpald forms a cyclic derivative with aldehyde, which upon oxidation turns from colorless to purple for which absorbance was read at 540 nm. Measurement of lens GSH levels was based on enzymatic recycling method [33, 34].

For nitrosative stress, lens nitrotyrosine (3-NT) levels, which indirectly provides estimation of ONOO^-^, were estimated using a commercially available sandwich-ELISA kit (Elabscience, Wuhan, China) containing wells pre-coated with antibody specific to 3-NT as per manufacturer’s instructions.

### Estimation of lens ATP contents

Lens ATP level was determined using a commercially available luminescence ATP assay kit (BioVision, CA, USA). Each lens was homogenized in the supplied reaction buffer in a ratio of 1 mg of tissue: 10 μl of reaction buffer. The homogenate was centrifuged at 890 g for 30 seconds at 4°C and supernatant was used for ATP measurement. 10 μl of standards or samples were added with 90 μl of reaction mix containing reaction buffer and enzyme mix into designated white-walled well plate. Luminescence was read using Victor X5 plate reader (Perkin Elmer, Waltham, MA).

### Estimation of lens Na^+^ K^+^ ATPase activity

Na^+^ K^+^ ATPase activity was determined by measuring the difference of inorganic phosphorus liberated in the presence or absence of ouabain as described by Khan et al. [35]. Briefly, lenses were homogenized in 50 mM Tris-HCl, pH 7.4 and 300 mM sucrose on ice, followed by centrifugation at 890 g at 4°C for 5 minutes. ATPase activity was determined in two reaction solutions, solution A and B. In solution A, tissue homogenate was added to 20 mM KCl, 100 mM NaCl, 5mM MgCl_2_ and 100 mM Tris-HCl followed by incubation at room temperature for 5 minutes. Reaction was started by adding 2.5 mM of ATP disodium salt and reaction mixture was incubated at 37°C for 15 minutes. In solution B, tissue homogenate was added to 5 mM MgCl_2_, 100 mM Tris-HCl and 1 mM ouabain (Na^+^ K^+^ ATPase specific inhibitor). The mixture was incubated at room temperature for 5 minutes before starting the reaction by adding 120 mM NaCl and 2.5 mM of ATP disodium salt followed by incubation at 37°C for 15 minutes. Reaction was terminated in both solution A and B by adding 10% trichloroacetic acid (TCA). Mixture was centrifuged at 890 g for 5 minutes and the supernatant was used for estimating inorganic phosphate. The activity of Na^+^ K^+^ ATPase was calculated as the difference of the inorganic phosphorus liberated between solution A and B.

To measure inorganic phosphate, supernatant was added with 2.5% ammonium molybdate (AM) reagent (2.5g of AM in 100 ml of 3N sulphuric acid) and developer reagent (0.5 g of 1-amino-2-napthol-4-sulphonic acid (ANSA) in 195ml of 15% of sodium metabisulphite and 5 ml of 20% of sodium sulphite). Mixture was vortexed and incubated for 10 minutes at room temperature. Absorbance was read at 640 nm. The enzyme activity was expressed as nanomoles of phosphorus liberated/min/mg protein.

### Estimation of Ca^2+^ ATPase activities

SERCA and PMCA activities were determined as described by Nagai et al. [36]. Lens tissue homogenate was prepared as described for Na^+^ K^+^ ATPase activity. For SERCA activity, solution containing 200 mM KCl, 100 mM HEPES, 10 mM MgCl_2_, 2 mM ethylene glycol tetraacetic acid (EGTA), 2mM ATP disodium salt and 2.2 mM CaCl2, pH 7.4, with or without 1μM thapsigargin (specific SERCA inhibitor) were added to tissue homogenate. The mixture was incubated for 1 hour at 37°C.

For PMCA activity, solution containing 200 mM KCl, 100 mM HEPES, 10 mM MgCl_2_, 2 mM EGTA, 2 mM ATP disodium salt and 1μM thapsigargin, pH 7.4, with or without 2.2 mM CaCl2 were added to tissue homogenate. The mixture was incubated for 1 hour at 37°C. The reaction was terminated by adding 10% TCA. The mixture was centrifuged at 890 g for 10 minutes at 4°C and the supernatant was used for inorganic phosphate determination as described in Na^+^ K^+^ ATPase activity. The activity of SERCA and PMCA was calculated as the difference of the inorganic phosphorus liberated in the presence or absence of thapsigargin or calcium.

### Estimation of calpain 2 activity and lens protein contents

Lens calpain 2 activity was measured using a commercially available sandwich-ELISA kit (Elabscience, Wuhan, China) containing wells pre-coated with antibody specific to calpain 2 as per manufacturer’s instructions.

Total protein level was determined using an aliquot of homogenized sample, before centrifugation. The homogenate was then centrifuged at 890 g for 15 minutes at 4°C and the supernatant was used for quantification of soluble protein. Lens protein level was determined using Bradford method, which detects change in the colour of Coomassie dye from brown to blue as a result of its binding to proteins in acidic medium.

### Statistical analysis

All numerical values were expressed as mean ± standard deviation (SD) while categorical variables expressed as frequency and percentage/bar chart. The difference of proportion of each cataract stage by intervention groups was tested using cross tabulation and Chi-square test. The difference in the mean weight, blood glucose and biochemical between the three intervention groups was analyzed using one-way ANOVA with post-hoc Bonferroni test. P value ≤ 0.05 was considered significant. IBM SPSS Statistics 24.0 was used to analyze all the data.

## Results

### Pilot study

**Fig 2:**
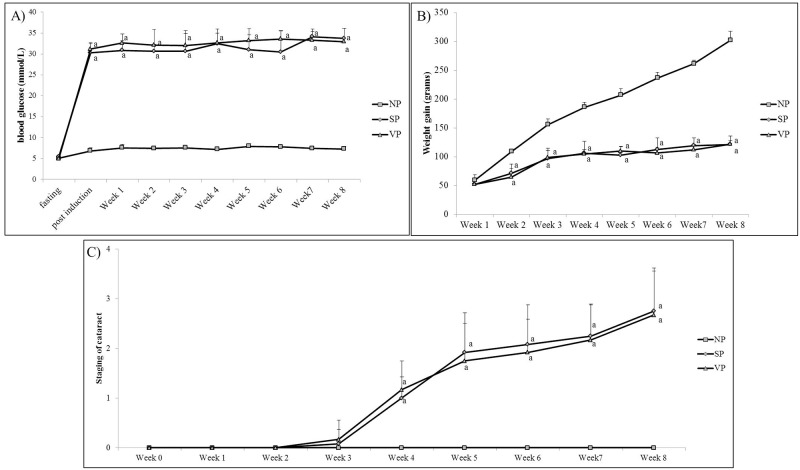
Pilot study showing A) weight gain (grams), B) blood glucose level (mmol/L) and C) cataract progression among 3 groups of rats. N = 6. ^a^p<0.001 versus NP. ANOVA with Bonferroni post-hoc indicated significant difference between NV against diabetic groups (SP and VP). NP: Normal rats, SP: Diabetic rats; VP: Diabetic rats with vehicle treatment.

Both STZ- induced diabetic groups (SP and VP) showed similar trend of body weight gain which was significantly lower compared to normal rats throughout the experimental period (Fig 2A). Similar level of hyperglycemia was observed in both the STZ- induced diabetic groups starting from 72 hours post STZ injection until the end of experimental period (Fig 2B). The cataractous changes observed in both STZ-induced diabetic groups also showed similar progression over the entire experimental period (Fig 2C).

### Main studies

#### Body weight and blood glucose level

**Fig 3:**
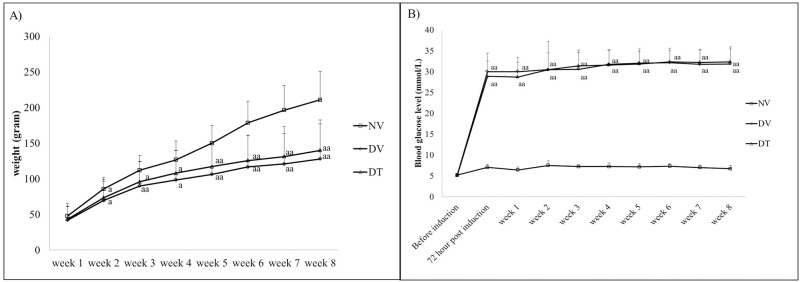
A) Weight gain (grams) and B) blood glucose level (mmol/L) among 3 groups of rats during 8 weeks of experimental period. N = 39. ^a^p<0.05 versus NV, ^aa^p<0.001 versus NV. ANOVA with Bonferroni post-hoc indicated significant difference between NV against diabetic groups (DV and DT). NV: Normal rats with vehicle treatment; DV: Diabetic rats with vehicle treatment; DT: Diabetic rats with tocotrienol treatment.

Both DV and DT groups showed significantly smaller weight gain compared to NV group from second week post-STZ injection until the end of experimental period. The blood glucose level in STZ injected rats showed significant increase 72 hours post-STZ injection and remained within the range of 27 to 34 mmol/L throughout the experimental period (p<0.001) in both the diabetic groups (Fig 3).

#### Anterior segment imaging and cataract grading

**Fig 4:**
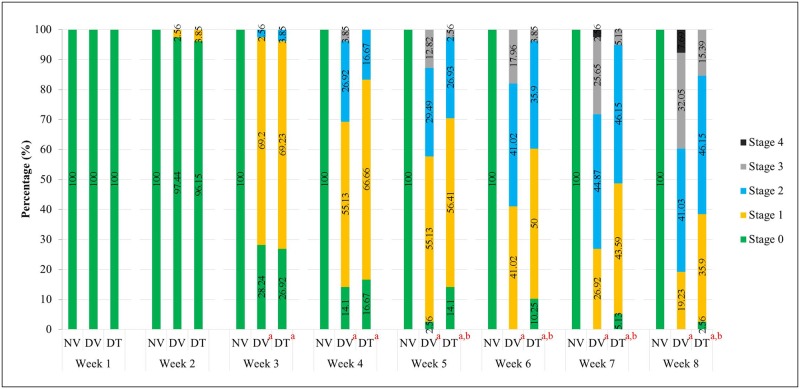
Effect of topically applied tocotrienol on development of cataract in STZ-induced diabetic rats during 8 weeks of experimental period. N = 78, ^a^p<0.001 versus NV; ^b^p<0.001 versus DV. NV: Normal rats with vehicle treatment; DV: Diabetic rats with vehicle treatment; DT: Diabetic rats with tocotrienol treatment.

Anterior segment imaging showed clear lenses in NV group until the end of experimental period (Fig 4). Onset of cataract in both the DV and DT groups started from 3rd week post-STZ injection and persisted until the end of treatment period. However, there was delayed cataract progression in DT group compared to DV group from 5^th^ week onward until the end of treatment period (p<0.001) (Fig 4) Additionally, we did not observe any local or systemic adverse effects throughout the experimental period.

#### Effect of tocotrienol on lenticular AR activity and polyol accumulation

Lenses in both the DV and DT groups showed significantly higher AR activity and sorbitol level compared to NV group. However, DT group showed 1.16-folds lower AR activity and 1.18-folds lower lens sorbitol level compared to DV group and the differences for both parameters were significant (Table 1).

**Table 1:** Effect of topically applied tocotrienol on lens aldose reductase and sorbitol activities in STZ-induced diabetic rats.

Group	Lens Aldose Reductase (ng/mg lens protein)	Lens Sorbitol (units/ g lens weight)
NV	2.19 ± 0.3	4.54 ± 0.2
DV	3.11 ± 0.3 ^a^	6.23 ± 0.4 ^a^
DT	2.69 ± 0.1 ^a^^,^^c^	5.28 ± 0.3 ^a^^,^^b^

N = 6, ANOVA with Bonferroni post-hoc indicated significant difference between NV-DV (mean difference: 0.92 & 1.69 respectively, p<0.001), NV-DT (mean difference: 0.49 & 0.74 respectively, p<0.01) and DV-DT (mean difference: 0.42 & 0.95, p<0.05 & p<0.001, respectively).

^a^ p<0.001 versus NV;

^b^ p<0.01 versus NV;

^c^ p<0.001 versus DV;

^d^ p<0.05 versus DV.

NV: Normal rats treated with vehicle; DV: Diabetic rats treated with vehicle; DT: Diabetic rats treated with tocotrienol

#### Effect of tocotrienol on lenticular pIκBα and NFκB activities

**Fig 5:**
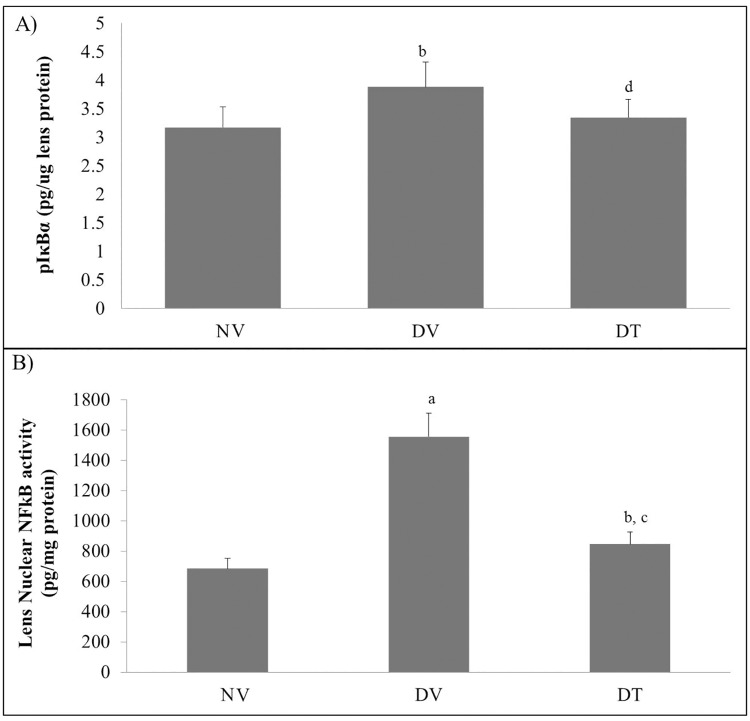
Effect of topically applied tocotrienol on lenticular A) pIκBα and B) NFκB in STZ-induced diabetic rats. N = 6, ANOVA with Bonferroni post-hoc indicated significant difference between NV-DV (mean difference: 0.71 & 871.80, p<0.05 & p<0.001 respectively) and DV-DT (mean difference: 0.53 & 709.65, p<0.05 & p< 0.001, respectively). ^a^p<0.001 versus NV; ^b^p<0.05 versus NV; ^c^p<0.001 versus DV; ^d^p<0.05 versus DV. NV: Normal rats treated with vehicle; DV: Diabetic rats treated with vehicle; DT: Diabetic rats treated with tocotrienol.

Quantitative measurement of pIκBα and NFκB by ELISA showed higher cytoplasmic pIκBα activity in DV group compared to DT and NV groups (p<0.05) whereas the same was comparable between DT and NV groups (Fig 5A). Nuclear NFκB activity was 2.28- and 1.24-folds higher in DV and DT groups, respectively, compared to group NV group. However, DT group showed 1.84-folds lesser NFκB activity compared to DV group (p<0.001) (Fig 5B).

**Fig 6:**
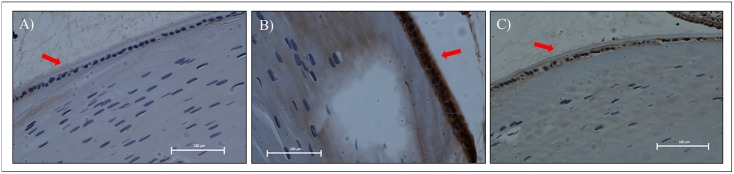
Tissue sections of rat lenses after immunostaining with NFκB p65 antibody in STZ-induced diabetic and normal rats. A- NV group showing normal epithelium with absence of brown stain for NFκB p65, B- DV group showing dense brown-stained lens epithelium, C- DT group showing minimal brown stain for NFκB p65 compared to DV group. Red arrows indicate lens epithelial layer. Scale bar represents 100μm. NV: Normal rats treated with vehicle; DV: Diabetic rats treated with vehicle; DT: Diabetic rats treated with tocotrienol.

Immunohistochemical staining for NFκB p65 was observed particularly in the lens epithelial and equatorial region. Positive activity was represented by brown-coloured stain that was mainly visible in the DV group and not in both the NV and DT groups (Fig 6).

#### Effect of tocotrienol on lenticular *iNOS* gene and protein expression

**Fig 7:**
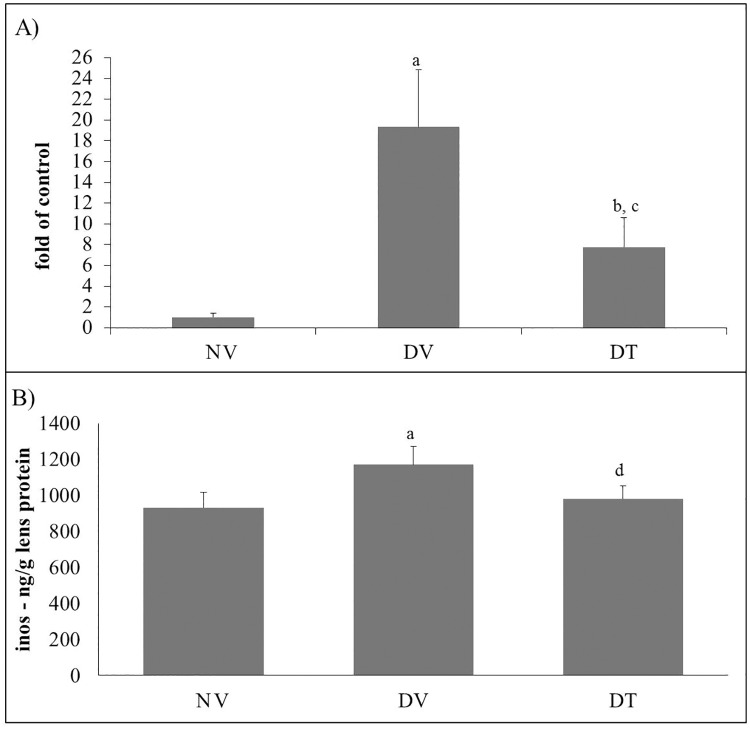
Effect of topically applied tocotrienol on lenticular A) *iNOS* gene expression and B) iNOS activity in STZ-induced diabetic rats. N = 6, ANOVA with Bonferroni post-hoc indicated significant difference between NV-DV (mean difference: 18.39 & 237.15 respectively, p<0.001) and DV-DT (mean difference: 11.56 & 188.39, p<0.001 & p<0.01, respectively). ^a^p<0.001 versus NV; ^b^p<0.05 versus NV; ^c^p<0.001 versus DV, ^d^p<0.01 versus DV. NV: Normal rats treated with vehicle; DV: Diabetic rats treated with vehicle; DT: Diabetic rats treated with tocotrienol.

iNOS mRNA expression in DV group was 19.2-folds higher compared to NV group (p<0.001). DT group also showed 7.7-folds higher iNOS mRNA expression compared to NV group (p<0.05). However, there was 2.5-folds lower expression of iNOS mRNA expression in DT compared DV group (p<0.001) (Fig 7A). iNOS protein expression as estimated by ELISA was higher in DV group by 1.25- and 1.19- folds compared to NV (p<0.001) and DT (p<0.01) groups, respectively. The iNOS protein in DT group, however, was comparable to NV group (Fig 7B).

#### Effect of tocotrienol on lenticular oxidative and nitrosative stress

We observed 1.27 folds higher level of lenticular MDA in DV group compared to NV group (p<0.001), whereas, DT group had 1.18 folds lower MDA content compared to DV group (p<0.001). Both catalase and SOD enzyme activities in DT group were comparable to that in NV group. The GSH levels were 3.63 and 1.72 folds lower in both DV and DT groups compared to NV group (p<0.001), respectively. However, DT group showed 1.25 fold higher GSH levels compared to DV group (p<0.01) (Table 2). DV group showed higher lenticular 3-NT activity compared to NV group (p<0.001), however, the same in DT group was comparable with that of NV group (Table 2).

**Table 2:** Effect of topically applied tocotrienol on lenticular MDA, GSH, catalase, SOD and 3-NT in STZ-induced diabetic rats.

Group	MDA (μM/g lens weight)	Catalase activity (μmol/g lens protein)	SOD activity (units/mg lens protein)	GSH level (μmol/g lens weight)	3-NT activity (ng/mg lens protein)
NV	903.29 ± 46.0	65.82 ± 11.4	9.27 ± 0.3	5.49 ± 0.3	8.62 ± 0.2
DV	1143.04 ± 89.6 ^a^	136.09 ± 24.4 ^a^	7.17 ± 0.8 ^a^	2.55 ± 0.1 ^a^	9.75 ± 0.5 ^a^
DT	967.26 ± 33.2 ^b^	65.44 ± 16.3 ^b^	9.74 ± 0.6 ^b^	3.19 ± 0.3 ^a^^,^^c^	8.62 ± 0.5 ^b^

N = 6, ANOVA with Bonferroni post-hoc indicated significant difference between NV-DV (mean difference: 239.75, 70.27, 2.11, 2.95 & 1.13 respectively, p<0.001) and DV-DT (mean difference: 175.78, 70.65, 2.57, 0.641 & 1.13; p<0.001, p<0.001, p<0.001, p<0.01 & p<0.001 respectively).

^a^ p<0.001 versus NV;

^b^ p<0.001 versus DV;

^c^ p<0.01 versus DV.

NV: Normal rats treated with vehicle; DV: Diabetic rats treated with vehicle; DT: Diabetic rats treated with tocotrienol

#### Effect of tocotrienol on lenticular ATP and ATPases

Lenticular ATP level in DT group was higher in trend compared to DV group and comparable to NV group. Lenticular Na^+^ K^+^ ATPase activity in DT group was restored to normal, whereas the DV group showed lower activity compared to NV and DT groups. Lenticular PMCA activity in DV group was lower compared to NV group (p<0.01), while the same in DT group was comparable to N group. Lenticular SERCA activity in DT group was 1.25-folds higher compared to DV group (Table 3).

**Table 3:** Effect of topically applied tocotrienol on lenticular ATP level and ATPases activity in STZ-induced diabetic rats.

Groups	ATP (pmol/g lens weight)	Na^+^ K^+^ ATPase activity (nmol Pi liberated/mg protein/ min)	PMCA activity (μmol Pi liberated/mg protein/ min)	SERCA activity (μmol Pi liberated/mg protein/ min)
NV	901.11 ± 79.2	2.73 ± 0.5	3.17 ± 0.3	1.66 ± 0.6
DV	804.21 ± 35.1	1.92 ± 0.7^a^	2.60 ± 0.3^a^	1.33 ± 0.2
DT	867.36 ± 51.3	2.63 ± 0.3	3.35 ± 0.3^b^	1.67 ± 0.3

N = 6, ANOVA with Bonferroni post-hoc indicated significant difference in Na^+^ K^+^ ATPase activity between NV-DV (mean difference: 0.81, p<0.05) and PMCA activity between NV-DV (mean difference: 0.57, p<0.05) and DV-DT (mean difference: 0.75; p<0.01).

^a^ p<0.05 versus NV;

^b^ p<0.01 versus DV.

NV: Normal rats treated with vehicle; DV: Diabetic rats treated with vehicle; DT: Diabetic rats treated with tocotrienol

#### Effect of tocotrienol on calpain 2 and lenticular protein levels

**Fig 8:**
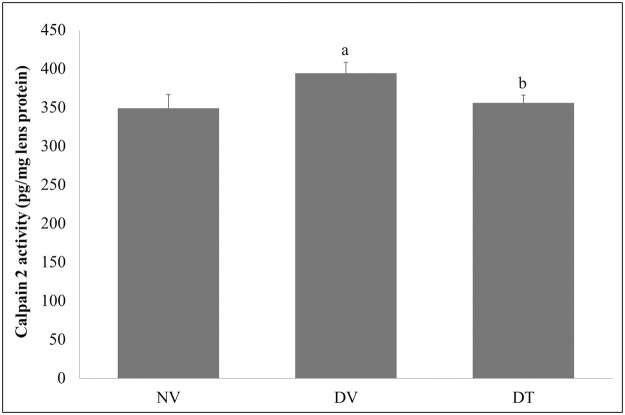
Effect of topically applied tocotrienol on lens calpain 2 activity. N = 6, ANOVA with Bonferroni post-hoc indicated significant difference in lens calpain between NV-DV (mean difference: 45.25, p<0.001) and DV-DT (mean difference: 38.68; p<0.001). ^a^p<0.001 versus NV; ^b^p<0.001 versus DV. NV: Normal rats with vehicle treatment; DV: Diabetic rats with vehicle treatment; DT: Diabetic rats with tocotrienol treatment.

The lens calpain 2 activity in DV group showed higher mean value compared to NV and DT groups (1.13- and 1.11-folds respectively; p<0.001) whereas, DT group showed no significant difference from NV group (Fig 8). The soluble to insoluble protein ratio was 1.4-folds lower in DV group compared to NV group (p<0.05). However, this ratio was restored to normal in DT group (Table 4).

**Table 4:** Effect of topically applied tocotrienol on lenticular proteins in STZ-induced diabetic rats.

Groups	Total protein (mg/g lens weight)	Soluble protein (mg/g lens weight)	Insoluble protein (mg/g lens weight)	Soluble:Insoluble protein (ratio)
NV	473.78 ± 22.3	309.54 ± 21.5	164.24 ± 28.3	1.93 ± 0.3
DV	490.24 ± 46.9	280.89 ± 11.2^a^	209.35 ± 39.0^a^	1.39 ± 0.3^a^
DT	468.83 ± 31.4	311.61 ± 9.8^b^	157.21 ± 23.5^b^	2.02 ± 0.3^b^

N = 6, ANOVA with Bonferroni post-hoc indicated significant difference between NV-DV (mean difference: 28.64, 45.11 & 0.54 respectively, p<0.05) and DV-DT (mean difference: 30.72, 52.13 & 0.63 respectively, p<0.05).

^a^ p<0.05 versus NV;

^b^ p<0.05 versus DV.

NV: Normal rats treated with vehicle; DV: Diabetic rats treated with vehicle; DT: Diabetic rats treated with tocotrienol

## Discussion

The current study demonstrated that topical application of the microemulsion formulation of tocotrienol delays the progression of cataract in rats with STZ-induced diabetes despite persistent hyperglycemia. The delayed cataractogenesis might be due to reduction of lenticular AR activity, polyol accumulation, NFκB expression and activation, iNOS expression, oxidative-nitrosative stress and calpain 2 activity resulting in improved soluble:insoluble protein ratio and delayed lenticular opacities development.

Complications of diabetes such as cataract have been attributed to long standing hyperglycemia [37] and the mechanisms that relate hyperglycemia with diabetic complications have widely been investigated. Since presence of AR as well as polyols has been detected in cataractous lenses [6, 38], polyol pathway seems to play a significant role. Polyol pathway involves two enzymes of which AR is the first one that converts glucose to sorbitol and the second enzyme, sorbitol dehydrogenase, converts sorbitol to fructose. However, the rate of conversion of sorbitol to fructose is considerably slow and sorbitol does not diffuse out of the cell, hence resulting in its intracellular accumulation [39]. The role of AR in cataractogenesis is further supported by other studies that observed rodents having low or no AR activity are resistant to development of diabetic cataract [40, 41]. Furthermore, Lee et al. [42] demonstrated that cataract development in type 2 diabetes mellitus patient may be influenced by polymorphism in AR gene. In the current study, reduced AR activity and polyol accumulation may be the key mechanism in the anticataract effect of tocotrienol. It is noteworthy that lenticular tocotrienol’s effects seen in this study was not secondary to reduction in blood glucose levels as orally administered tocotrienol has been reported to reduce blood glucose level in STZ-induced diabetic rat [43]. Both tocotrienol- and vehicle-treated diabetic group showed similar persistent hyperglycemia and trends in terms of weight gain throughout the study, as demonstrated by earlier studies [44–46].

Increased AR activity is positively correlated with oxidative stress primarily by causing NADPH depletion. In this regards, Lou et al. [47] demonstrated restoration of GSH level to near normal with administration of AR inhibitor. Polyphenols including tocopherol, the other analogue of vitamin E, have been shown to possess AR inhibitory activity [48–50], however, the same has not been demonstrated for tocotrienol. Direct aldose reductase inhibition assay may provide evidence if tocotrienol directly inhibits AR. Nevertheless, the reduced oxidative stress in response to treatment with tocotrienol in our study may be attributed to reduce polyol pathway activity as been observed reduced AR activity and polyol accumulation in tocotrienol-treated rats. Tocotrienol, however, may also scavenge ROS by donating phenolic hydrogen to free radicals [51]. Reduced lenticular oxidative stress in tocotrienol treated group in this study was in accordance with that observed in galactose-fed rats in our previous study [31].

ROS have been shown to act as secondary signaling messengers that activate many phosphosignaling pathways including NFκB [52]. Exposure of human lens epithelial cells (HLECs) to ultraviolet radiations was shown to exacerbate ROS production which plays an essential role in the activation of NFκB [53]. Furthermore, this activation of NFκB in HLECs involves phosphorylation of IκB-α [54]. NFκB exists in cytoplasm complexed with IκB-α and phosphorylation of IκB-α releases NFκB which translocates to nucleus and modulates transcription of several genes including iNOS in the lens [55]. Lower nuclear NFκB and cytoplasmic phosphorylated IκB-α levels in the tocotrienol treated group compared to vehicle treated group indicated reduced NFκB activation. This is in accordance with other studies which showed ability of tocotrienol to inhibit NFκB activation in vitro [56–59] as well as in vivo [60,61]. It has been suggested that inhibition of NFκB activation by tocotrienol may be attributed to decreased IκB phosphorylation due to suppression of proteasomic activity [60–63]. Furthermore, Wang et al. [58] and Jiang et al. [59] demonstrated the ability of tocotrienol to modulate sphingolipid metabolism and that was shown to cause higher expression and activity of NFκB negative regulator in cytokine-induced RAW 264.7 macrophages. The NFκB inhibitory effect was previously demonstrated for γ- and δ-tocotrienol [64] and the annatto tocotrienol used in this study consist of both γ and δ isomer [65].

Activation and migration of NFκB to the nucleus in the presence of hyperglycemia leads to its increased binding to the iNOS promoter and a consequent increase in iNOS expression [66]. Increased iNOS expression is positively correlated with cataractogenesis and Inomata et al. [55] detected increased expressions of iNOS mRNA and iNOS protein in cataractous lenses. Since we observed reduced iNOS expression in the lenses of animals treated with tocotrienol, it is likely that reduced NFκB expression secondary to reduced ROS generation was the contributory factor. We demonstrated that topical tocotrienol not only affects iNOS protein expression, but also reduced its gene expression. Furthermore, this present study also showed reduced nitrotyrosine levels in tocotrienol treated rats also indicate reduced NO production due to reduced iNOS expression. Additionally, Inomata et al. [55] also showed the induction of iNOS protein occurs before the elevation in calcium content and increase in calpain-mediated proteolysis, both of which are closely related to the development of lens opacification.

This present study also observed that induction of diabetes causes depletion of lens ATP levels. Mitton et al. [67] have shown earlier that in STZ-induced diabetic rats lenticular ATP/ADP ratio begin to decrease one week post-induction, even before the onset of cataractous changes. ATP loss may be due to oxidative stress [68] and mitochondrial damage [69] that leads to reduced expression of cytochrome c oxidase, an enzyme important in mitochondrial respiratory pathway for ATP synthesis [70]. We observed normalization of lens ATP contents following tocotrienol treatment which was in accordance with similar observation made by Schloesser et al [71] using brain of aged mice and Nowak et al. [72] using renal proximal tubular cells. Thus, the preservation of lenticular ATP following treatment with tocotrienol in the current study may be attributed to its potent antioxidant effects. Furthermore, effect of tocotrienol on maintaining ATP levels may also be due to its ability to preserve mitochondrial function in the presence of oxidative stress [72] and inhibition of cytochrome c release [73].

As the consequence of preservation of lens ATP levels, lens Na^+^ K^+^ ATPase and Ca^2+^ ATPase activity in tocotrienol treated groups were restored. However, loss of ATPase functions may also be a consequence of oxidative stress. Wang et al. [74] have shown that Na^+^ K^+^ ATPase activity is highly sensitive to changes in ATP and ROS levels. Huang et al. [75] showed modification of Na^+^ K^+^ ATPase is due to oxidative damage. Similarly Ca^2+^ ATPase activity is affected by oxidative stress [76]. Borchman et al. [77] showed that Ca^2+^ ATPase activity could be inhibited through oxidation in membrane-enriched sample from rabbit cortical and epithelial lens fibers treated with hydrogen peroxide. Moreover, oxidative damage to the lipid membranes may cause the uncoupling of membrane bound ATPases, thus reducing their activity [78, 79]. It is likely that tocotrienol associated restoration of ATPase functions involves reduction of lens oxidative stress besides correction of lens ATP levels. Additionally, vitamin E and tocotrienol have also been shown to normalize the oxidative damage-induced reduction in Na^+^ K^+^ ATPase activity in carbofuran-treated rat [80] and tert-butylhydroperoxide-treated human erythrocytes [81].

Na^+^ K^+^ ATPase has long been recognized for its role in regulating electrolyte concentrations in the lens, which is vital to lens transparency. Dysfunction of Na^+^ K^+^ ATPase leads to elevated lens Na^+^ level which promotes lens opacification [24]. Similarly, maintenance of calcium homeostasis is imperative for the clarity of the lens. Ca^2+^ ATPase is essential for the removal of cytosolic calcium, either across the plasma membrane or through intracellular organelles such as the endoplasmic reticulum [82]. The Ca^2+^ ATPase activity in the membranes of cataractous lenses was shown to be 50% lesser compared to normal lenses [83]. Hence, in the current study, restoration of Na^+^ K^+^ ATPase and Ca^2+^ ATPase activity in response to treatment with tocotrienol seems to play a key role in maintaining the lenticular ionic homeostasis. This was also evidenced by reduced calpain 2 activity and normalized soluble: insoluble protein ratio in tocotrienol treated diabetic rats in this study. Since the activity of calpain is calcium-dependent, it is evident that increased lenticular Ca^2+^ subsequent to Ca^2+^ ATPase dysfunction promotes lens opacification by causing proteolysis of soluble lens proteins into insoluble ones [76].

In conclusion, current study demonstrated that topical application of tocotrienol prevents the progression of cataract in STZ-induced diabetic rats in the presence of persistent hyperglycemia. This delayed cataractogenesis could be attributed to reduced AR activation and reduced polyol accumulation leading to reduced lenticular oxidative stress. Tocotrienol also reduces lens nitrosative stress due to reduced iNOS expression as a consequence of reduced NFκB activation. Additionally, tocotrienol preserves lens ATP and restores Na^+^ K^+^ ATPase and Ca^2+^ ATPase activity and consequently reduces calpain activity, resulting in restoration of soluble: insoluble protein ratio and delayed cataractogenesis. Further studies to determine the extent of ocular penetration of tocotrienol, and not only preventive but therapeutic anticataract effects of tocotrienol would determine the potential of tocotrienol as an anticataract agent.

## Supporting information

S1 Table Blood sugar data for all experimental groups. (PDF)

S2 Table Weight data for all experimental groups. (PDF)

S3 Table Cataract staging data for all experimental groups. (PDF)

S4 Table Blood parameters data for all experimental groups. (PDF)
