# Supplementary material for: Reduction of oxidative-nitrosative stress underlies anticataract effect of topically applied tocotrienol in streptozotocin-induced diabetic rats
Source: PLoS One. 2017 Mar 28;12(3):e0174542. doi: 10.1371/journal.pone.0174542 (PMC5370128; doi:10.1371/journal.pone.0174542)
Supplement: S1 Table — (PDF) [file pone.0174542.s001.pdf]

Blood sugar

Group	FBS before induce	RBS post induce	Week 1	Week 2	Week 3	Week 4	Week 5	Week 6	Week 7	Week 8
N	4.8	7	7	7	7.4	7.4	8.2	7.5	7.9	7.9
N	5	7	7	6.9	7	8.4	7.7	7.9	6.7	6.6
N	6	7	7	7.2	7	7.9	8	7.9	7.4	7.4
N	5	6.8	5.2	7.1	6.8	6.5	6.7	6.6	6.8	5.2
N	4.9	8	5.8	9.9	8	6.8	6.5	8.3	6.6	6.7
N	5.6	8	6.3	6.5	8	7.8	6.8	7	7.5	7
N	5	5.6	6.8	8.6	6.7	5.6	6	6.2	6	6.3
N	4.8	7	7	7	7.4	7.4	8.2	7.5	7.9	7.9
N	5	7	7	6.9	7	8.4	7.7	7.9	6.7	6.6
N	6	7	7	7.2	7	7.9	8	7.9	7.4	7.4
N	5	6.8	5.2	7.1	6.8	6.5	6.7	6.6	6.8	5.2
N	4.9	8	5.8	9.9	8	6.8	6.5	8.3	6.6	6.7
N	5.6	8	6.3	6.5	8	7.8	6.8	7	7.5	7
N	5	5.6	6.8	8.6	6.7	5.6	6	6.2	6	6.3
N	4.8	7	7	7	7.4	7.4	8.2	7.5	7.9	7.9
N	5	7	7	6.9	7	8.4	7.7	7.9	6.7	6.6
N	6	7	7	7.2	7	7.9	8	7.9	7.4	7.4
N	5	6.8	5.2	7.1	6.8	6.5	6.7	6.6	6.8	5.2
N	4.9	8	5.8	9.9	8	6.8	6.5	8.3	6.6	6.7
N	5.6	8	6.3	6.5	8	7.8	6.8	7	7.5	7
N	5	5.6	6.8	8.6	6.7	5.6	6	6.2	6	6.3
N	4.8	7	7	7	7.4	7.4	8.2	7.5	7.9	7.9
N	5	7	7	6.9	7	8.4	7.7	7.9	6.7	6.6
N	6	7	7	7.2	7	7.9	8	7.9	7.4	7.4
N	5	6.8	5.2	7.1	6.8	6.5	6.7	6.6	6.8	5.2
N	4.9	8	5.8	9.9	8	6.8	6.5	8.3	6.6	6.7
N	5.6	8	6.3	6.5	8	7.8	6.8	7	7.5	7

N	5	5.6	6.8	8.6	6.7	5.6	6	6.2	6	6.3
N	4.8	7	7	7	7.4	7.4	8.2	7.5	7.9	7.9
N	5	7	7	6.9	7	8.4	7.7	7.9	6.7	6.6
N	6	7	7	7.2	7	7.9	8	7.9	7.4	7.4
N	5	6.8	5.2	7.1	6.8	6.5	6.7	6.6	6.8	5.2
N	4.9	8	5.8	9.9	8	6.8	6.5	8.3	6.6	6.7
N	5.6	8	6.3	6.5	8	7.8	6.8	7	7.5	7
N	5	5.6	6.8	8.6	6.7	5.6	6	6.2	6	6.3
N	4.8	7	7	7	7.4	7.4	8.2	7.5	7.9	7.9
N	5	7	7	6.9	7	8.4	7.7	7.9	6.7	6.6
N	6	7	7	7.2	7	7.9	8	7.9	7.4	7.4
N	5	6.8	5.2	7.1	6.8	6.5	6.7	6.6	6.8	5.2
DV	5.2	32.6	35	22.6	27	29.1	30	28.5	27.9	25.5
DV	4.8	31.5	29.9	23.3	26.6	25.7	27	26.4	30.3	27.2
DV	4.6	24	26.6	28.5	29	29	23	29	23.5	26.5
DV	5.1	33.2	28.9	30.7	28.8	23.3	35	35	26.1	26.3
DV	4.9	30.1	30.7	35	31.2	31.2	30	35	35	31.7
DV	5.6	31	31.1	31.6	16.6	23	35	35	30.9	35
DV	6	35	25.8	24	33	35	29.4	23.2	35	33.2
DV	6.6	22.6	28.7	28.3	30.5	30.7	24.8	35	29.7	30.9
DV	4.8	26.4	31.1	30	22.7	23	32.5	25.8	35	26
DV	5	30.5	32.3	31.5	35	35	33.6	31.8	32.9	35
DV	5	22.5	28.9	33	35	34	30.7	32.8	27	35
DV	5	30	31.4	28.1	32.2	33.2	35	33.8	27.9	28.5
DV	4.9	30.9	32.4	35	35	35	35	35	35	35
DV	5	29.9	29.4	35	30.3	32.6	30.9	31.9	35	35
DV	4.9	30.1	35	35	35	35	35	35	35	35
DV	4.8	27	31.1	26	27.9	31.1	35	30.6	27.2	27.1
DV	5.2	32	30.5	35	35	35	35	35	35	35
DV	4.8	23.9	28.7	24.1	24.1	28	25	29	35	29.5

DV	5	27.4	29.4	28.3	35	33.7	33.2	31.3	26.4	24.8
DV	5.4	35	27.3	27.6	27.3	28.2	32.5	32.6	31.6	33
DV	5.6	35	35	33	34	31.6	29.6	31.8	32.8	28.6
DV	4.8	20.6	27.9	31.5	29.8	28.6	28.9	31.6	28	28.7
DV	5	26.2	27.6	24.5	33.5	35	33.7	35	35	35
DV	5.2	24.9	33	30.3	23.6	29	25.9	29.6	30.1	27.8
DV	5	28.6	35	26.4	26.5	32.1	31.3	25	28.8	32.7
DV	5.3	33	30.2	30	22.4	35	31.8	27.5	32	35
DV	5.6	35	34	30.6	35	32.1	35	35	33.4	35
DV	5.1	35	35	35	30.3	35	29.2	35	35	35
DV	5.2	35	31.9	35	35	34.1	35	35	35	35
DV	5.3	35	28	35	35	32.1	35	33.8	31.1	28.5
DV	5.4	35	28.7	34.3	33.9	35	35	35	35	35
DV	5.6	35	31.4	30.6	31.6	35	35	35	30.6	35
DV	5.4	35	26.5	33.6	32.6	35	35	35	35	35
DV	4.8	35	33.9	33.5	33.5	35	35	35	35	35
DV	4.9	23	23.3	24.2	35	32	33.2	32.5	28.2	30.3
DV	4.7	28.1	29.3	35	35	31.2	32.2	35	35	35
DV	5.3	23.4	18.1	25.9	27.5	30.8	31.6	25.1	28.2	30.7
DV	4.9	30.5	31.6	35	35	35	35	35	35	35
DV	4.7	31.2	26.5	33.1	27.2	35	35	35	35	35
DT	5	32.9	29.6	22.8	35	25	28	29.4	27.5	35
DT	5	32.7	33	23.9	35	33.1	35	28.3	28.5	28.2
DT	4.9	35	24.4	32.2	27.4	27.2	31.7	29	31.2	28.6
DT	5	25.9	25.9	35	26.5	30.1	35	30.6	34	33
DT	5.6	32.5	30.7	25	25	35	31.5	30.5	31.7	27.9
DT	4.8	27.7	24.5	25.9	31.4	35	30.1	35	26	35
DT	4.8	28.6	29.9	33.2	35	25.2	33.2	35	29.6	35
DT	5	35	30.7	33.2	35	35	35	35	35	35
DT	5.2	30.6	26.5	25	25.2	29.3	32.2	35	30	21.3

DT	6	28.9	31.2	30.4	35	30.7	29.7	35	35	35
DT	4.5	30.8	30.6	33.8	35	33.6	33.4	34	31	35
DT	5	32.6	30.1	28	35	30.1	33.5	35	32.6	35
DT	5.2	24.1	25	27.2	29.1	31.2	32.1	32.6	35	27.9
DT	5	24.2	17.3	23.6	32.9	25.1	31.1	30.5	35	35
DT	4.7	27	25.9	27	26.8	32.2	29.2	31.2	31	30.4
DT	4.8	31.1	29.5	35	29.5	30	30.7	29	26.1	28.6
DT	4.9	30	29.1	32.7	31.1	29.8	31.5	32.4	35	35
DT	4.8	27.1	26.4	35	32	33.2	27.3	31	31.5	28.5
DT	4.9	24.1	33.1	31.2	35	35	34.1	35	35	35
DT	5	25.5	31	35	35	35	35	32.5	35	33.4
DT	5.2	25.6	32.2	25.2	31	31	27.1	27.9	32.5	31.3
DT	5	24.4	21.9	21	32.7	28.6	32	28.3	33.8	32.5
DT	5.3	25.9	31.1	24.8	22.9	21.9	31.7	27.4	23.7	24.6
DT	5.6	35	31.6	63.9	32.9	33.8	33.5	35	35	35
DT	5	35	32.5	29.3	31	33.5	29.7	35	35	35
DT	4.9	28.8	31	30.2	31.2	28	25.4	33.5	32.5	35
DT	4.8	25.8	26.9	28.4	30.4	34	32.9	35	35	35
DT	5	31.6	29.9	29.2	33.1	35	35	33.7	35	33.6
DT	5	31.1	25.1	29	29.7	31.4	23.9	30.9	29.1	31.2
DT	4.9	23.9	32.7	30.7	31.5	31.8	35	31.9	30.2	35
DT	4.9	23.9	29.7	33.7	28.5	32.8	33.9	32.8	31.2	32.5
DT	4.8	27.1	26.4	35	32	33.2	27.3	31	31.5	28.5
DT	5	27.1	28.6	26.8	30.2	35	32.1	26.7	28	26
DT	5.4	35	33.5	30.4	35	35	35	35	35	35
DT	5.5	24.8	31	35	35	35	35	35	35	35
DT	5	31.1	31	32.5	31.1	35	35	35	35	35
DT	5	31.5	23.3	25.5	31.3	28.1	31.2	31.7	35	35
DT	5.3	28.5	30.5	35	32	35	35	35	35	35
DT	4.8	25.8	26.9	28.4	30.4	34	32.9	35	35	35
