# Supplementary material for: Reduction of oxidative-nitrosative stress underlies anticataract effect of topically applied tocotrienol in streptozotocin-induced diabetic rats
Source: PLoS One. 2017 Mar 28;12(3):e0174542. doi: 10.1371/journal.pone.0174542 (PMC5370128; doi:10.1371/journal.pone.0174542)
Supplement: S2 Table — (PDF) [file pone.0174542.s002.pdf]

Weight

Group	Week 1	Week 2	Week 3	Week 4	Week 5	Week 6	Week 7	Week 8
N	70	110	123	174	175	198	230	260
N	70	90	110	152	132	200	210	220
N	65	84	104	130	139	204	214	229
N	50	104	154	104	154	191	203	213
N	29	88	123	88	123	160	182	191
N	30	74	109	140	186	200	219	240
N	29	72	101	139	191	219	254	280
N	38	68	72	94	128	128	150	155
N	56	104	127	130	140	146	152	170
N	37	90	125	153	171	196	218	221
N	28	50	85	101	130	140	143	150
N	71	83	104	108	121	143	160	172
N	70	110	123	174	175	198	230	260
N	70	90	110	152	132	200	210	220
N	65	84	104	130	139	204	214	229
N	50	104	154	104	154	191	203	213
N	29	88	123	88	123	160	182	191
N	30	74	109	140	186	200	219	240
N	29	72	101	139	191	219	254	280
N	38	68	72	94	128	128	150	155
N	56	104	127	130	140	146	152	170
N	37	90	125	153	171	196	218	221
N	28	50	85	101	130	140	143	150
N	71	83	104	108	121	143	160	172
N	70	110	123	174	175	198	230	260
N	70	90	110	152	132	200	210	220
N	65	84	104	130	139	204	214	229
N	50	104	154	104	154	191	203	213
N	29	88	123	88	123	160	182	191
N	30	74	109	140	186	200	219	240
N	29	72	101	139	191	219	254	280
N	38	68	72	94	128	128	150	155
N	56	104	127	130	140	146	152	170
N	30	88	110	120	150	197	210	217
N	60	105	127	140	166	188	204	215
N	58	99	130	145	178	183	202	223
N	43	70	100	115	134	169	190	210
N	35	70	105	120	135	175	195	202
N	57	88	100	118	126	159	180	201

DV	21	13	21	8	51	76	89	76
DV	16	93	113	143	143	145	151	165
DV	16	93	116	136	188	211	220	233
DV	39	53	116	118	126	118	132	129
DV	20	101	132	132	80	105	132	90
DV	25	102	130	148	158	158	168	188
DV	87	120	136	130	115	153	105	146
DV	38	98	145	161	171	193	194	231
DV	38	75	93	88	100	86	117	130
DV	21	59	95	52	106	126	114	126
DV	74	90	133	149	171	187	193	177
DV	47	83	104	115	69	137	118	129
DV	38	39	50	42	39	61	57	59
DV	55	55	103	58	122	126	129	142
DV	45	78	91	125	125	74	114	68
DV	75	77	97	114	109	121	139	151
DV	78	98	118	149	169	151	69	90
DV	87	106	96	116	136	149	146	152
DV	29	30	42	35	49	43	32	45
DV	37	72	91	99	113	102	107	140
DV	38	79	99	122	124	130	144	146
DV	20	28	38	38	38	50	68	65
DV	40	58	57	79	71	83	90	60
DV	29	56	77	69	63	63	109	111
DV	38	66	77	86	76	77	96	103
DV	19	33	59	66	40	77	86	102
DV	35	99	122	141	147	155	168	182
DV	51	95	130	137	144	146	167	184
DV	48	76	74	88	114	158	135	140
DV	36	46	56	50	60	79	76	92
DV	31	24	49	44	63	73	69	89
DV	46	67	54	66	78	69	95	88
DV	61	45	90	130	144	174	182	208
DV	55	80	84	108	125	142	140	154
DV	52	77	103	112	112	117	114	138
DV	27	43	25	31	31	43	52	48
DV	63	114	153	159	162	168	182	193
DV	37	54	89	97	108	119	114	118
DV	26	39	61	102	105	109	98	107
DT	34	56	77	76	94	101	101	101
DT	39	93	155	172	160	187	226	227

DT	32	78	104	97	103	138	137	155
DT	23	92	121	134	138	154	159	190
DT	35	80	125	137	160	165	185	201
DT	44	89	111	125	148	180	192	205
DT	40	98	136	89	135	142	159	173
DT	72	101	97	127	130	134	124	163
DT	45	70	125	155	166	174	185	204
DT	20	49	57	70	75	90	61	75
DT	73	110	135	135	143	149	142	142
DT	69	79	80	114	96	101	55	84
DT	64	110	116	148	148	149	147	133
DT	38	77	77	104	131	122	124	124
DT	69	104	115	121	135	148	174	182
DT	65	84	108	118	126	109	129	98
DT	68	80	103	132	142	126	141	126
DT	36	87	115	130	144	117	134	147
DT	59	78	130	153	167	191	187	201
DT	57	106	124	135	151	166	176	181
DT	38	54	95	106	120	114	136	130
DT	20	39	78	117	108	123	131	133
DT	53	98	105	120	115	138	142	163
DT	35	75	101	116	122	136	152	155
DT	16	34	40	40	54	55	52	62
DT	39	60	86	101	114	110	116	155
DT	24	84	122	123	147	155	167	194
DT	37	57	78	96	117	132	145	134
DT	62	67	65	75	96	104	110	124
DT	56	74	83	88	101	96	121	122
DT	43	54	69	77	76	98	90	109
DT	10	12	39	47	66	58	73	90
DT	28	55	75	86	67	68	85	105
DT	13	38	46	66	60	65	66	85
DT	51	75	82	79	64	94	84	86
DT	65	97	96	107	129	139	133	100
DT	57	96	119	145	158	163	164	162
DT	22	7	57	38	49	83	84	98
DT	44	41	33	35	70	80	95	99
