# Supplementary material for: Reduction of oxidative-nitrosative stress underlies anticataract effect of topically applied tocotrienol in streptozotocin-induced diabetic rats
Source: PLoS One. 2017 Mar 28;12(3):e0174542. doi: 10.1371/journal.pone.0174542 (PMC5370128; doi:10.1371/journal.pone.0174542)
Supplement: S3 Table — (PDF) [file pone.0174542.s003.pdf]

Staging data

Group	Week 1	Week 2	Week 3	Week 4	Week 5	Week 6	Week 7	Week 8	Week 9
N	0	0	0	0	0	0	0	0	0
N	0	0	0	0	0	0	0	0	0
N	0	0	0	0	0	0	0	0	0
N	0	0	0	0	0	0	0	0	0
N	0	0	0	0	0	0	0	0	0
N	0	0	0	0	0	0	0	0	0
N	0	0	0	0	0	0	0	0	0
N	0	0	0	0	0	0	0	0	0
N	0	0	0	0	0	0	0	0	0
N	0	0	0	0	0	0	0	0	0
N	0	0	0	0	0	0	0	0	0
N	0	0	0	0	0	0	0	0	0
N	0	0	0	0	0	0	0	0	0
N	0	0	0	0	0	0	0	0	0
N	0	0	0	0	0	0	0	0	0
N	0	0	0	0	0	0	0	0	0
N	0	0	0	0	0	0	0	0	0
N	0	0	0	0	0	0	0	0	0
N	0	0	0	0	0	0	0	0	0
N	0	0	0	0	0	0	0	0	0
N	0	0	0	0	0	0	0	0	0
N	0	0	0	0	0	0	0	0	0
N	0	0	0	0	0	0	0	0	0
N	0	0	0	0	0	0	0	0	0
N	0	0	0	0	0	0	0	0	0
N	0	0	0	0	0	0	0	0	0
N	0	0	0	0	0	0	0	0	0
N	0	0	0	0	0	0	0	0	0
N	0	0	0	0	0	0	0	0	0
N	0	0	0	0	0	0	0	0	0
N	0	0	0	0	0	0	0	0	0
N	0	0	0	0	0	0	0	0	0
N	0	0	0	0	0	0	0	0	0
N	0	0	0	0	0	0	0	0	0
N	0	0	0	0	0	0	0	0	0
N	0	0	0	0	0	0	0	0	0
N	0	0	0	0	0	0	0	0	0
N	0	0	0	0	0	0	0	0	0

N	0	0	0	0	0	0	0	0	0
N	0	0	0	0	0	0	0	0	0
N	0	0	0	0	0	0	0	0	0
N	0	0	0	0	0	0	0	0	0
N	0	0	0	0	0	0	0	0	0
N	0	0	0	0	0	0	0	0	0
N	0	0	0	0	0	0	0	0	0
N	0	0	0	0	0	0	0	0	0
N	0	0	0	0	0	0	0	0	0
N	0	0	0	0	0	0	0	0	0
N	0	0	0	0	0	0	0	0	0
N	0	0	0	0	0	0	0	0	0
N	0	0	0	0	0	0	0	0	0
N	0	0	0	0	0	0	0	0	0
N	0	0	0	0	0	0	0	0	0
N	0	0	0	0	0	0	0	0	0
N	0	0	0	0	0	0	0	0	0
N	0	0	0	0	0	0	0	0	0
N	0	0	0	0	0	0	0	0	0
N	0	0	0	0	0	0	0	0	0
N	0	0	0	0	0	0	0	0	0
N	0	0	0	0	0	0	0	0	0
N	0	0	0	0	0	0	0	0	0
N	0	0	0	0	0	0	0	0	0
N	0	0	0	0	0	0	0	0	0
N	0	0	0	0	0	0	0	0	0
N	0	0	0	0	0	0	0	0	0
N	0	0	0	0	0	0	0	0	0
N	0	0	0	0	0	0	0	0	0
N	0	0	0	0	0	0	0	0	0
N	0	0	0	0	0	0	0	0	0
N	0	0	0	0	0	0	0	0	0
N	0	0	0	0	0	0	0	0	0
N	0	0	0	0	0	0	0	0	0
N	0	0	0	0	0	0	0	0	0
N	0	0	0	0	0	0	0	0	0
N	0	0	0	0	0	0	0	0	0
N	0	0	0	0	0	0	0	0	0
N	0	0	0	0	0	0	0	0	0
N	0	0	0	0	0	0	0	0	0
DV	0	0	0	1	2	2	3	3	3

DV	0	0	0	1	2	2	3	3	4
DV	0	0	0	0	0	1	1	1	2
DV	0	0	0	0	1	1	2	2	2
DV	0	0	0	0	0	0	1	1	1
DV	0	0	0	0	0	0	1	1	1
DV	0	0	0	1	1	1	1	1	1
DV	0	0	0	1	1	1	1	1	1
DV	0	0	0	1	1	1	1	2	3
DV	0	0	0	1	1	1	1	1	1
DV	0	0	0	1	2	2	2	2	2
DV	0	0	0	1	2	2	2	2	2
DV	0	0	0	1	1	1	1	1	1
DV	0	0	0	1	1	1	1	2	2
DV	0	0	0	0	1	1	1	1	2
DV	0	0	0	0	1	1	2	2	2
DV	0	0	0	0	1	1	1	1	1
DV	0	0	0	1	1	1	1	1	1
DV	0	0	0	1	3	3	3	3	3
DV	0	0	0	1	3	3	3	3	4
DV	0	0	0	1	1	1	1	1	1
DV	0	0	0	1	1	1	1	1	1
DV	0	0	0	1	2	3	3	3	3
DV	0	0	0	1	2	3	3	3	3
DV	0	0	0	0	0	1	1	2	2
DV	0	0	0	1	1	1	2	2	2
DV	0	0	0	0	0	2	2	2	2
DV	0	0	0	0	1	2	2	2	2
DV	0	0	0	0	1	1	2	2	2
DV	0	0	0	0	1	1	1	2	2
DV	0	0	1	2	2	2	2	2	3
DV	0	0	1	2	2	2	2	2	2
DV	0	0	0	1	1	1	2	2	2
DV	0	0	0	1	1	1	2	2	3
DV	0	0	0	1	2	3	3	3	3
DV	0	0	0	1	2	2	2	2	3
DV	0	0	0	0	1	1	1	1	1
DV	0	0	0	1	1	1	1	1	1
DV	0	0	0	0	1	1	2	3	3
DV	0	0	0	1	1	1	1	2	2
DV	0	0	0	1	2	3	3	3	3
DV	0	0	0	1	1	1	1	1	1

DV	0	0	0	1	3	3	3	3	3
DV	0	0	0	1	1	2	3	3	3
DV	0	0	0	0	0	2	2	2	2
DV	0	0	0	0	0	2	2	3	3
DV	0	0	0	1	1	1	1	2	3
DV	0	0	0	1	1	1	1	2	2
DV	0	0	0	1	2	3	3	3	3
DV	0	0	0	1	1	2	2	2	2
DV	0	0	0	1	1	1	2	3	4
DV	0	0	0	1	1	1	2	3	3
DV	0	0	0	1	1	1	1	1	1
DV	0	0	0	1	1	1	1	1	1
DV	0	0	0	0	0	1	1	2	2
DV	0	0	0	0	0	1	1	2	3
DV	0	0	0	0	1	1	1	3	3
DV	0	0	0	0	1	1	1	2	2
DV	0	0	0	1	1	1	1	1	3
DV	0	0	0	1	1	1	1	1	2
DV	0	0	0	0	0	1	2	2	2
DV	0	0	0	0	0	1	2	2	2
DV	0	0	0	1	2	2	2	2	3
DV	0	0	0	1	2	2	2	2	3
DV	0	0	0	1	1	1	2	2	2
DV	0	0	0	1	1	1	2	2	2
DV	0	0	0	1	1	1	1	1	2
DV	0	0	0	1	1	1	1	1	2
DV	0	0	0	1	1	2	2	3	4
DV	0	0	0	1	1	2	2	3	3
DV	0	0	0	1	2	3	3	4	4
DV	0	0	0	1	2	2	3	4	4
DV	0	0	0	1	2	2	2	2	2
DV	0	0	0	1	2	2	2	2	2
DV	0	0	0	1	2	2	2	3	3
DV	0	0	0	1	2	3	3	3	3
DV	0	0	0	1	1	2	2	2	2
DV	0	0	0	1	2	2	2	2	2
DT	0	0	0	1	1	1	1	1	2
DT	0	0	0	1	1	1	1	2	2
DT	0	0	0	1	1	1	2	2	2
DT	0	0	0	1	2	2	2	2	2
DT	0	0	0	1	1	1	1	1	1

DT	0	0	0	1	1	1	1	1	1
DT	0	0	0	1	1	1	1	1	1
DT	0	0	0	1	1	1	1	1	1
DT	0	0	0	0	0	1	1	2	2
DT	0	0	0	0	0	0	1	2	2
DT	0	0	1	2	2	2	2	2	2
DT	0	0	1	2	2	2	2	2	2
DT	0	0	0	1	1	2	3	3	3
DT	0	0	1	2	2	2	2	2	2
DT	0	0	0	1	1	1	1	1	1
DT	0	0	0	1	1	1	1	1	1
DT	0	0	0	1	1	1	1	2	2
DT	0	0	0	1	1	1	1	2	2
DT	0	0	0	1	2	3	3	3	3
DT	0	0	0	1	2	2	2	2	2
DT	0	0	0	1	2	3	3	3	3
DT	0	0	0	1	1	2	2	2	2
DT	0	0	0	1	1	2	2	2	2
DT	0	0	0	1	1	1	2	2	3
DT	0	0	0	1	1	1	1	2	2
DT	0	0	0	1	1	1	1	2	2
DT	0	0	0	1	1	1	2	2	3
DT	0	0	0	1	1	1	2	2	3
DT	0	0	0	1	1	1	1	1	1
DT	0	0	0	1	1	1	1	1	1
DT	0	0	0	1	1	2	2	2	2
DT	0	0	0	1	2	2	2	2	2
DT	0	0	0	0	0	0	0	0	0
DT	0	0	0	0	0	0	1	1	1
DT	0	0	0	0	1	1	1	1	1
DT	0	0	0	0	1	1	1	1	1
DT	0	0	0	1	1	2	2	2	2
DT	0	0	0	0	2	2	2	3	3
DT	0	0	0	0	1	1	1	1	1
DT	0	0	0	0	0	0	0	0	0
DT	0	0	0	1	1	1	1	1	3
DT	0	0	0	1	2	2	2	2	2
DT	0	0	0	1	1	1	2	2	2
DT	0	0	0	1	2	2	2	2	2
DT	0	0	0	1	1	1	1	1	1
DT	0	0	0	1	1	1	1	1	1

DT	0	0	0	0	1	1	1	1	2
DT	0	0	0	0	1	1	1	1	1
DT	0	0	0	0	0	0	0	0	2
DT	0	0	0	0	1	1	1	1	1
DT	0	0	0	0	0	0	0	1	1
DT	0	0	0	0	0	0	0	1	1
DT	0	0	0	0	0	1	1	1	1
DT	0	0	0	0	0	0	1	1	1
DT	0	0	0	1	1	1	1	1	1
DT	0	0	0	1	1	1	1	1	2
DT	0	0	0	0	0	0	0	1	2
DT	0	0	0	0	0	0	0	1	3
DT	0	0	0	1	1	1	1	1	1
DT	0	0	0	1	1	1	1	2	2
DT	0	0	0	1	1	2	2	2	2
DT	0	0	0	1	1	2	2	2	2
DT	0	0	0	1	1	2	2	2	3
DT	0	0	0	1	1	2	2	2	2
DT	0	0	0	1	1	1	2	2	2
DT	0	0	0	1	1	1	1	1	3
DT	0	0	0	0	1	1	1	1	1
DT	0	0	0	0	0	0	0	0	1
DT	0	0	0	1	1	1	1	1	1
DT	0	0	0	1	1	2	2	2	2
DT	0	0	0	1	1	1	1	1	1
DT	0	0	0	1	1	1	1	1	1
DT	0	0	0	1	2	2	2	2	2
DT	0	0	0	1	2	2	2	2	2
DT	0	0	0	1	1	1	2	2	2
DT	0	0	0	1	1	1	1	1	1
DT	0	0	0	1	1	1	2	2	3
DT	0	0	0	1	1	1	1	2	2
