# Supplementary material for: Reduction of oxidative-nitrosative stress underlies anticataract effect of topically applied tocotrienol in streptozotocin-induced diabetic rats
Source: PLoS One. 2017 Mar 28;12(3):e0174542. doi: 10.1371/journal.pone.0174542 (PMC5370128; doi:10.1371/journal.pone.0174542)
Supplement: S4 Table — (PDF) [file pone.0174542.s004.pdf]

| | Parameters | | | | | | | | | | | | | |
|---|---|---|---|---|---|---|---|---|---|---|---|---|---|---|
| Group | Aldose Reductase (ng/mg lens protein) | Sorbitol (units/g lens weight) | pIκBα (pg/ug lens protein) | NFκB (pg/mg protein) | iNOS (ng/g lens protein) | MDA (μM/g lens weight) | Catalase (μmol/g lens protein) | SOD (units/mg lens protein) | GSH (μmol/g lens weight) | 3-NT (ng/mg lens protein) | ATP (pmol/g lens weight) | $Na^+$ $K^+$ ATPase activity (nmol Pi liberated/mg protein/min) | PMCA activity (μmol Pi liberated/mg protein/min) | SERCA activity (μmol Pi liberated/mg protein/min) |
| N | 2.1912 | 4.2464 | 2.4745 | 622.2552 | 989.7800 | 896.9156 | 62.9653 | 9.8444 | 5.5421 | 8.6194 | 850.5354 | 3.2218 | 2.8585 | 1.1680 |
| N | 2.2798 | 4.6379 | 3.2504 | 694.1662 | 901.3981 | 954.8105 | 60.6244 | 9.2441 | 4.9838 | 8.1597 | 837.0385 | 2.4562 | 3.2956 | 1.3211 |
| N | 1.8864 | 4.7815 | 3.5378 | 675.4436 | 952.0816 | 917.7741 | 84.5971 | 8.9862 | 5.5836 | 8.6352 | 1044.9496 | 2.6746 | 3.1490 | 2.1203 |
| N | 1.9459 | 4.2176 | 3.1569 | 815.1200 | 1011.8126 | 931.7460 | 70.8862 | 9.4015 | 5.3635 | 8.7951 | 884.5004 | 3.3702 | 3.6656 | 1.0644 |
| N | 2.5881 | 4.6181 | 3.2380 | 632.3922 | 779.5538 | 897.8405 | 65.4495 | 9.0138 | 5.7972 | 8.6839 | 852.0639 | 2.1067 | 2.7982 | 1.8298 |
| N | 2.2803 | 4.7233 | 3.3548 | 662.6788 | 954.7450 | 820.6349 | 50.3992 | 9.1498 | 5.6860 | 8.8284 | 937.5866 | 2.5413 | 3.2662 | 2.4323 |
| DV | 2.8616 | 5.8028 | 3.8661 | 1575.4917 | 1117.2938 | 1042.2932 | 116.5535 | 6.8786 | 2.4952 | 9.3252 | 823.4424 | 2.0180 | 2.3824 | 1.3994 |
| DV | 3.1989 | 6.4745 | 4.2833 | 1312.1585 | 1135.5036 | 1095.9184 | 151.5145 | 8.0365 | 2.3199 | 10.6683 | 799.8207 | 1.2729 | 2.8120 | 1.0763 |
| DV | 3.2620 | 6.5470 | 4.5040 | 1426.8300 | 1359.7064 | 1175.7519 | 147.9707 | 6.5711 | 2.5254 | 9.5050 | 786.3904 | 1.4870 | 2.9916 | 1.1996 |
| DV | 3.5233 | 6.7842 | 3.6379 | 1618.2637 | 1140.0622 | 1195.4365 | 122.1227 | 6.4217 | 2.7476 | 9.5275 | 862.8942 | 1.3187 | 2.2380 | 1.5906 |
| DV | 2.9458 | 6.1098 | 3.6100 | 1706.6441 | 1072.5924 | 1069.4981 | 107.5179 | 6.7350 | 2.6726 | 9.4702 | 785.9385 | 2.7868 | 2.5573 | 1.5816 |
| DV | 2.8885 | 5.6562 | 3.3715 | 1693.4409 | 1187.0829 | 1279.3367 | 170.8338 | 8.3472 | 2.5202 | 9.9858 | 766.1785 | 2.6498 | 2.6188 | 1.1264 |
| DT | 2.8286 | 5.6043 | 3.6795 | 866.8727 | 909.8309 | 960.9635 | 59.5391 | 9.7934 | 2.7954 | 9.0585 | 936.7578 | 2.7172 | 3.1001 | 2.0250 |
| DT | 2.4671 | 5.5625 | 3.0475 | 928.4775 | 884.4320 | 988.9456 | 86.3037 | 10.6292 | 2.8743 | 8.2924 | 788.6712 | 2.8609 | 3.7275 | 1.9027 |
| DT | 2.6368 | 5.5281 | 2.9517 | 868.7069 | 983.5257 | 955.7823 | 47.420835 | 8.8786 | 3.0519 | 8.4767 | 903.0221 | 3.0407 | 2.8474 | 1.7419 |
| DT | 2.7502 | 5.0925 | 3.5308 | 789.0014 | 995.3909 | 974.2525 | 84.50257 | 9.7510 | 3.5119 | 9.2355 | 855.7748 | 2.2995 | 3.4149 | 1.4243 |
| DT | 2.6896 | 4.8982 | 3.6464 | 905.5971 | 1052.0660 | 1010.5519 | 53.207227 | 9.8803 | 3.6627 | 8.6645 | 859.7247 | 2.3437 | 3.5704 | 1.5104 |
| DT | 2.7618 | 4.9997 | 3.2095 | 716.2488 | 1056.6498 | 913.0435 | 61.655192 | 9.4827 | 3.2326 | 7.9703 | 839.8591 | 2.5192 | 3.4332 | 1.4366 |

| Group | Calpain (pg/mg lens protein) | Total protein (mg/g lens weight) | Soluble protein (mg/g lens weight) | Insoluble protein (mg/g lens weight) | Soluble: Insolubl e protein (ratio) |
|---|---|---|---|---|---|
| N | 315.6393 | 479.3190 | 305.9653 | 173.3537 | 1.7650 |
| N | 356.3861 | 460.4858 | 319.5935 | 140.8923 | 2.2684 |
| N | 359.8263 | 486.6766 | 289.5130 | 197.1636 | 1.4684 |
| N | 358.3222 | 502.0816 | 346.1946 | 194.4900 | 1.7800 |
| N | 359.7063 | 437.7156 | 307.5916 | 149.3428 | 2.0596 |
| N | 348.0058 | 476.3902 | 288.3728 | 130.1956 | 2.2149 |
| DV | 385.8401 | 439.5317 | 259.1055 | 228.9214 | 1.1319 |
| DV | 420.9828 | 424.3661 | 278.5644 | 225.8354 | 1.2335 |
| DV | 396.8950 | 508.6407 | 286.8102 | 253.2660 | 1.1324 |
| DV | 390.2426 | 516.0187 | 287.0973 | 180.4262 | 1.5912 |
| DV | 383.1912 | 510.9830 | 285.1476 | 145.8017 | 1.9557 |
| DV | 392.2591 | 541.8900 | 288.6239 | 221.8306 | 1.3011 |
| DT | 344.5403 | 447.3568 | 309.5972 | 137.7596 | 2.2474 |
| DT | 360.4892 | 505.9502 | 306.3030 | 191.5610 | 1.5990 |
| DT | 345.0266 | 425.8095 | 314.3891 | 129.5430 | 2.4269 |
| DT | 369.2331 | 487.6583 | 296.2665 | 164.6654 | 1.7992 |
| DT | 354.9786 | 452.5783 | 322.9929 | 146.2753 | 2.2081 |
| DT | 363.0694 | 493.5989 | 320.1280 | 173.4709 | 1.8454 |
